# Efficacy and safety of acupuncture-point stimulation combined with opioids for the treatment of moderate to severe cancer pain: a network meta-analysis of randomized controlled trials

**DOI:** 10.3389/fonc.2023.1166580

**Published:** 2023-06-02

**Authors:** Qinglin Zhang, Yuntong Yuan, Meiling Zhang, Baohua Qiao, Yiyuan Cui, Ying Wang, Li Feng

**Affiliations:** ^1^ Department of Oncology, The Third Clinical Medical College, Zhejiang Chinese Medical University, Hangzhou, China; ^2^ Dongzhimen Hospital, Beijing University of Chinese Medicine, Beijing, China; ^3^ National Cancer Center/National Clinical Research Center for Cancer/Cancer Hospital, Chinese Academy of Medical Sciences and Peking Union Medical College, Beijing, China

**Keywords:** cancer pain, acupuncture-point stimulation, opioid, supplementary alternative therapy, network meta-analysis

## Abstract

**Background:**

Pain is one of the most common and troublesome symptoms of cancer. Although potential positive effects of acupuncture-point stimulation (APS) on cancer pain have been observed, knowledge regarding the selection of the optimal APS remains unclear because of a lack of evidence from head-to-head randomized controlled trials (RCTs).

**Objective:**

This study aimed to carry out a network meta-analysis to compare the efficacy and safety of different APS combined with opioids in treating moderate to severe cancer pain and rank these methods for practical consideration.

**Methods:**

A comprehensive search of eight electronic databases was conducted to obtain RCTs involving different APS combined with opioids for moderate to severe cancer pain. Data were screened and extracted independently using predesigned forms. The quality of RCTs was appraised with the Cochrane Collaboration risk-of-bias tool. The primary outcome was the total pain relief rate. Secondary outcomes were the total incidence of adverse reactions, the incidence of nausea and vomiting, and the incidence of constipation. We applied a frequentist, fixed-effect network meta-analysis model to pool effect sizes across trials using rate ratios (RR) with their 95% confidence intervals (CI). Network meta-analysis was performed using Stata/SE 16.0.

**Results:**

We included 48 RCTs, which consisted of 4,026 patients, and investigated nine interventions. A network meta-analysis showed that a combination of APS and opioids was superior in relieving moderate to severe cancer pain and reducing the incidence of adverse reactions such as nausea, vomiting, and constipation compared to opioids alone. The ranking of total pain relief rates was as follows: fire needle (surface under the cumulative ranking curve (SUCRA) = 91.1%), body acupuncture (SUCRA = 85.0%), point embedding (SUCRA = 67.7%), auricular acupuncture (SUCRA = 53.8%), moxibustion (SUCRA = 41.9%), transcutaneous electrical acupoint stimulation (TEAS) (SUCRA = 39.0%), electroacupuncture (SUCRA = 37.4%), and wrist–ankle acupuncture (SUCRA = 34.1%). The ranking of total incidence of adverse reactions was as follows: auricular acupuncture (SUCRA = 23.3%), electroacupuncture (SUCRA = 25.1%), fire needle (SUCRA = 27.2%), point embedding (SUCRA = 42.6%), moxibustion (SUCRA = 48.2%), body acupuncture (SUCRA = 49.8%), wrist–ankle acupuncture (SUCRA = 57.8%), TEAS (SUCRA = 76.3%), and opioids alone (SUCRA = 99.7%).

**Conclusions:**

APS seemed to be effective in relieving cancer pain and reducing opioid-related adverse reactions. Fire needle combined with opioids may be a promising intervention to reduce moderate to severe cancer pain as well as reduce opioid-related adverse reactions. However, the evidence was not conclusive. More high-quality trials investigating the stability of evidence levels of different interventions on cancer pain must be conducted.

**Systematic Review Registration:**

https://www.crd.york.ac.uk/PROSPERO/#searchadvanced, identifier CRD42022362054.

## 1 Introduction

Cancer is the world’s second leading cause of death, and its incidence rate increased yearly due to population aging and unhealthy lifestyle. Global cancer cases are estimated to reach 28.4 million by 2040, which is a 47% increase over 2020 (1). Pain is one of the most common and intractable symptoms of cancer caused by tumors or antitumor treatments and can occur at various stages of tumors. This is an important reason for patients to have their life quality decreased and to lose their confidence in treatment (2). According to statistics, about 40% of early to intermediate cancer patients and 90% of terminal cancer patients have suffered from moderate to severe cancer pain, of which 70% have not been effectively controlled (3). Opioids are the primary drugs used to treat moderate to severe cancer pain, but up to two-thirds of cancer patients reported inadequate pain management (4). Moreover, opioids may cause unexpected side effects, including nausea, vomiting, constipation, sedation, and cognitive impairment. Due to their inability to tolerate these adverse events, 10% to 20% of patients stop taking drugs (2).

In order to relieve cancer pain and reduce the demand for painkillers, various nondrug methods have been adopted, including acupuncture-point stimulation (APS), educational intervention, and relaxation. APS has been considered a promising alternative analgesia (5). In recent years, APS analgesia has been widely studied and generally recognized as safe, effective, and easy to operate with few adverse reactions (6–8). The role of APS in controlling cancer pain has been gradually verified (9, 10). Apart from relieving pain, APS could also help relieve fatigue and improve the quality of life for patients receiving palliative treatment (11). The National Comprehensive Cancer Network Adult Cancer Pain Guidelines have included acupuncture as a comprehensive intervention for cancer pain (2).

However, APS is diverse and has different therapeutic advantages (12, 13). Existing original studies and meta-analyses mostly compare APS combined with opioid therapy and opioids alone, lacking comparison among different APS methods in opioid-used situations. Therefore, this study used network meta-analysis to compare the efficacy and safety of different APS therapies in treating cancer pain. These outcome indexes were ranked quantitatively to find the best intervention for patients with moderate to severe cancer pain, which can provide evidence for selection prescription and medical decision-making.

## 2 Materials and methods

### 2.1 Search strategy

Four English-language databases (PubMed, Web of Science, EMBASE, Cochrane Central Register of Controlled Trials) and four Chinese-language databases (Chinese Biomedical Literature Database, China National Knowledge Infrastructure, VIP Database for Chinese Technical Periodicals, Wan Fang) were searched from the inception date to 30 June 2022. The search strategy consisted of three components: population (“cancer,” “tumor/tumor,” “carcinoma,” “neoplasm,” “pain,” “analgesia”), interventions (“acupuncture,” “electroacupuncture,” “manual acupuncture,” “moxibustion,” “point embedding,” “transcutaneous electrical acupoint stimulation,” “auricular point,” “thumb-tack acupuncture,” “wrist-ankle acupuncture,” “warm acupuncture”), and study type (“randomized clinical trial”). Existing systematic reviews were examined to identify additional trials. There were no restrictions on languages. Details of the search strategy are listed in **Supplementary Data 1**.

### 2.2 Inclusion criteria

Studies were included if they matched the following criteria: (1) Study type: randomized controlled trial, RCT. (2) Population: meet the diagnostic criteria of cancer pain (2); gender, race, and type of cancer are not limited. (3) Interventions: the control group was treated with opioids recommended by the WHO (combined with a placebo). On the basis of the control group, the experimental group was combined with APS therapy (filiform acupuncture, electroacupuncture, fire acupuncture, moxibustion, acupoint application, massage, and auricular acupuncture), and each therapy needs to be supported by sufficient research data (including RCTs ≥ 3, participants in each group ≥ 30). (4) Outcomes: at least one outcome measure. The primary outcome was the total pain relief rate. The main reference criteria were as follows (14): the cases of patients with partial relief or above (≥ 50%) or with marked effect or above were regarded as effective cases (i.e., the pain was tolerable and did not affect normal life or sleep). The safety outcomes were the incidence of adverse reactions (i.e., nausea/vomiting, constipation). We recorded the outcomes as close to 2 weeks as possible for all analyses. If the information at 2 weeks was not available, we used data ranging between 1 and 4 weeks and gave preference to the timepoint closest to 2 weeks. If time points were equidistant (i.e., 1 and 3 weeks), we took the longer outcome (3 weeks).

### 2.3 Exclusion criteria

(1) Studies with more than two APS methods combined interventions. (2) Studies on nonrandomized controlled trials or unbalanced baseline data between groups. (3) Studies with outcomes that cannot fit the design of this study. (4) Studies in which the data cannot be integrated, such as incorrect data or incomplete information. (5) Repeatedly published studies: only include one with the most complete data.

### 2.4 Literature screening and data extraction

All records found were imported into EndNote X9 to eliminate duplicate studies. Two independent reviewers (Q.L. Z. and Y.T. Y.) screened all titles and abstracts. Full-text articles of studies identified as potentially relevant were obtained and assessed by two independent reviewers according to the inclusion criteria. Only studies that met the inclusion criteria were selected for the systematic review and data extraction for network meta-analysis evaluation. Data were extracted from the included studies, including author name, published year, sample size, age, methods and duration of intervention, total pain relief rate, and adverse reactions. Any discrepancies were resolved by discussion or through adjudication by a third investigator (L. F.).

### 2.5 Risk-of-bias assessment

Two researchers (Q.L. Z. and Y.T. Y.) evaluated the risk of bias in each study based on the criteria of the revised Cochrane risk-of-bias tool (15). The quality evaluation items of each study included selection bias (random sequence generation and allocation concealment), performance bias (blinding of participants and personnel), detection bias (blinding of outcome assessment), attrition bias (incomplete outcome data), reporting bias (selective reporting), and other biases. These items were scored as low, high, or unclear risk of bias. Any discrepancies were resolved by consensus.

### 2.6 Data statistics and analysis

All analyses were performed using a network suite of commands in Stata/SE (version 16.0). The network package performed the network meta-analysis based on the frequentist framework (16). Firstly, network plots were drawn to show the quantitative relationship between various interventions. When it could form a closed loop, inconsistencies were detected. Statistical heterogeneity was investigated with the *I* ^2^ statistics and predefined heterogeneity (*I* ^2 =^ 0 indicates that the inconsistency of the results makes no statistical difference, *I* ^2^ ≤ 50% for low and *I* ^2^ > 50% for high). If *I*² ≤ 50%, a fixed-effects model was used; otherwise, a random-effects model was used. Next, the rate ratio (RR) with a 95% confidence interval (CI) was used to estimate the effect size. The results of the network meta-analysis were summarized based on all possible pairwise comparisons, including mixed comparisons and indirect comparisons. The effect of different interventions was estimated based on the surface under the cumulative ranking curve (SUCRA), which ranged from 0% to 100%. Synthetic sorting bubble diagrams based on the SUCRA value were drawn to comprehensively present the relatively better interventions in this Network Meta-Analysis (NMA). Finally, funnel plots were drawn to evaluate the publication bias and small samples of the included studies.

This review was conducted according to the guidelines of Preferred Reporting Items for Systematic Reviews and Meta-Analyses (17, 18). The protocol for this study was registered in the PROSPERO international prospective register of systematic reviews by the National Institute for Health Research (NIHR). The protocol registration ID is CRD42022362054.

## 3 Results

### 3.1 Literature search

**Figure 1:**
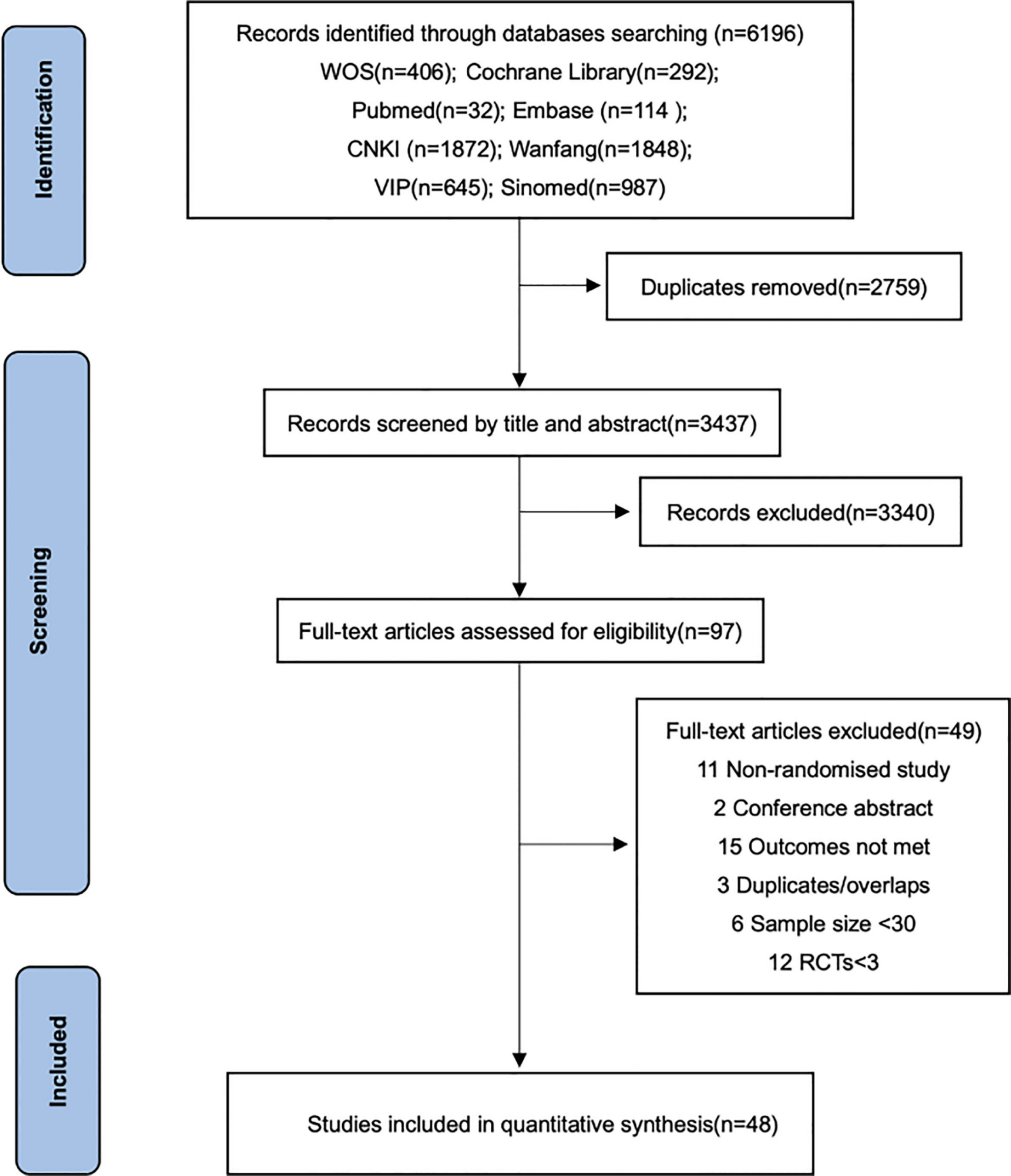
PRISMA flow diagram.

We identified 6,196 studies from the databases and trial registries and selected 97 possible eligible citations for full-text review. After excluding 49 studies, 48 trials met the inclusion criteria. The process of study search, screening, and selection is shown in **Figure 1**. These studies were all conducted in China, published between 2000 and 2022, and included a total of 4,026 patients (2,012 patients in the experiment group and 2,014 patients in the control group). All studies were two-arm studies with opioids as the intervention measures in the control group and APS combined with opioids in the experiment group.

Eight types of APS were reported among all trials. The main treatments were body acupuncture combined with opioids (19–30), moxibustion combined with opioids (31–38), electroacupuncture combined with opioids (39–43), auricular acupuncture combined with opioids (44–51), point embedding combined with opioids (52–54), transcutaneous electrical acupoint stimulation (TEAS) combined with opioids (55–60), wrist–ankle acupuncture combined with opioids (61–63), and fire needle combined with opioids (64–67). The number of research reporting pain relief was 45, while 29 studies mentioned treatment and painkiller-related adverse reactions (**Supplementary Data 2**).

Regarding the random sequence generation method, 30 studies used a random number list, two used simple randomization, and one used drawing lots to divide groups, which was evaluated as low risk. In total, 13 studies did not mention the specific randomization and were rated as unknown risks. Two studies were randomly assigned to the patient’s sequence of entry into the hospital and were classified as high-risk. All studies that did not mention allocation concealment were rated as unknown risks. All experimental groups were treated with APS on the basis of the control group. Although the blind method was mentioned in one study, it could not achieve blinding for the implementers and was rated as high risk. One study mentioned using blind methods for evaluators and statistical analysts and was rated as low risk, while other studies did not mention it and were all rated as unknown risks. All studies had complete data, so the attrition bias was evaluated as low risk. The proposals for all studies were not available and were rated as unknown risks. Other biases were unknown risks because there were no available details to evaluate (**Supplementary Figure S1**). Details of the risk of bias items across all included studies are listed in **Supplementary Figure S2**.

### 3.2 Consistency and heterogeneity analysis

No closed loop was formed in these outcomes, so the consistency model was directly selected. The heterogeneity of all outcome indicators was small at the overall level (*I*² < 50%), and the fixed-effect model was used for analysis.

### 3.3 Total pain relief rate

**Figure 2:**
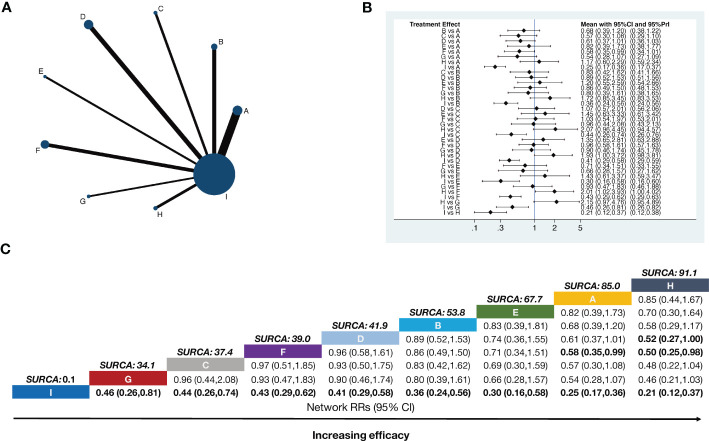
Network meta-analysis of total pain relief rate. The bold font indicates a statistically significant difference between the two treatments. **(A)** Network plot showing comparisons in efficacy between nodes (blue circles), each representing a unique intervention. Each node’s size is proportional to the total number of randomly assigned participants receiving the treatment. The width of each connecting line is proportional to the number of trial-level comparisons between the two nodes. **(B)** Forest plot of the network meta-analysis comparing the efficacy of each treatment. **(C)** Schematic detailing the most efficacious treatments according to the surface under the cumulative ranking curve analysis (SUCRA). **A,** Body acupuncture combined with opioids; **B,** moxibustion combined with opioids; **C,** electroacupuncture combined with opioids; **D,** auricular acupuncture combined with opioids; **E,** point embedding combined with opioids; **F,** TEAS combined with opioids; **G,** wrist–ankle acupuncture combined with opioids; **H,** fire needle combined with opioids; **I,** opioids.

A total of 45 two-arm studies referred to the total pain relief rate involving eight APS therapies. There were 11 studies on body acupuncture combined with opioids, six studies on moxibustion combined with opioids, four studies on electroacupuncture combined with opioids, eight studies on auricular acupuncture combined with opioids, three studies on point embedding combined with opioids, six studies on TEAS combined with opioids, three studies on wrist–ankle acupuncture combined with opioids, and four studies on fire needle combined with opioids. Using opioids as the comparison, eight pairs of direct comparisons were generated, and no closed loop was formed. A network of comparisons between interventions is shown in **Figure 2A**.

Compared with opioids alone, body acupuncture combined with opioids (RR = 0.25, 95% CI [0.17–0.36]), moxibustion combined with opioids (RR *=* 0.36, 95% CI [0.24–0.56]), electroacupuncture combined with opioids (RR *=* 0.44, 95% CI [0.26–0.74]), auricular acupuncture combined with opioids (RR *=* 0.41, 95% CI [0.29–0.58]), point embedding combined with opioids (RR *=*0.30, 95% CI [0.16–0.58]), TEAS combined with opioids (RR *=* 0.43, 95% CI [0.29–0.62]), wrist–ankle acupuncture combined with opioids (RR *=* 0.46, 95% CI [0.26–0.81]), and fire needle combined with opioids (RR *=* 0.21, 95% CI [0.12–0.37]) could improve the total pain relief rate and make the difference between groups statistically significant. Body acupuncture (RR *=* 0.58, 95% CI [0.35–0.99]) and auricular acupuncture (RR *=* 0.50, 95% CI [0.25–0.98]) showed a more practical function than TEAS in relieving pain, all of them happened in opioid-used situation. The RR values are shown in **Figure 2B**.

The SUCRA rank and probability value results indicated that fire needle combined with opioids (91.1%) was most likely to improve the pain relief rate, followed by body acupuncture combined with opioids (85.0%), point embedding combined with opioids (67.7%), auricular acupuncture combined with opioids (53.8%), moxibustion combined with opioids (41.9%), TEAS combined with opioids (39.0%), electroacupuncture combined with opioids (37.4%), wrist–ankle acupuncture combined with opioids (34.1%), and opioids alone (0.1%) (**Figure 2C**).

### 3.4 The safety of APS combined with opioids

#### 3.4.1 The total incidence of adverse reactions

**Figure 3:**
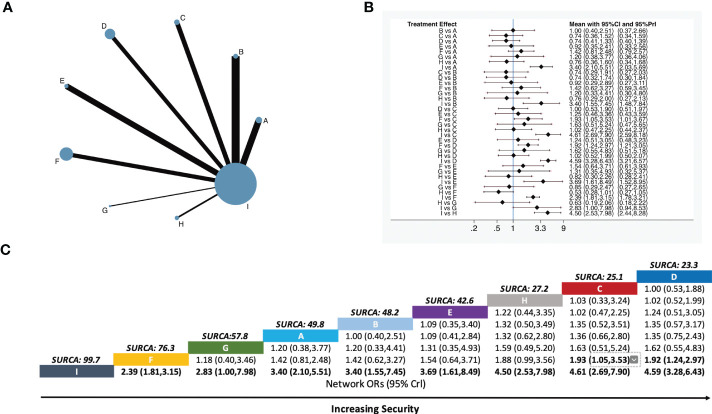
Network meta-analysis of total incidence of the adverse reactions. **(A)** Network plot showing comparisons in security between each intervention. **(B)** Forest plot of the network meta-analysis comparing the security of each treatment. **(C)** Schematic detailing the safest treatments according to the surface under the cumulative ranking curve analysis (SUCRA). **A,** Body acupuncture combined with opioids; **B,** moxibustion combined with opioids; **C,** electroacupuncture combined with opioids; **D,** auricular acupuncture combined with opioids; **E,** point embedding combined with opioids; **F,** TEAS combined with opioids; **G,** wrist–ankle acupuncture combined with opioids; **H,** fire needle combined with opioids; **I,** opioids.

The occurrence of adverse reactions was noted in 29 studies, making up eight pairs of direct comparisons. The adverse reactions mainly include dizziness, headache, dysuria, abdominal discomfort, diarrhea, nausea, vomiting, poor appetite, and drowsiness. There were six studies on body acupuncture combined with opioids, four studies on moxibustion combined with opioids, four studies on electroacupuncture combined with opioids, four studies on auricular acupuncture combined with opioids, three studies on point embedding combined with opioids, five studies on TEAS combined with opioids, one study on wrist–ankle acupuncture combined with opioids, and two studies on fire needle combined with opioids. No closed loop was formed in terms of safety outcome indicators. The network diagram is shown in **Figure 3A**.

Eight-pair comparisons were generated among the nine interventions. Compared with opioids alone, body acupuncture combined with opioids (RR *=* 3.40, 95% CI [2.10–5.51]), moxibustion combined with opioids (RR *=* 3.40, 95% CI [1.55–7.45]), electroacupuncture combined with opioids (RR *=* 4.61 95% CI [2.69–7.90]), auricular acupuncture combined with opioids (RR *=* 4.59, 95% CI [3.28–6.43]), point embedding combined with opioids (RR *=* 3.69, 95% CI [1.61–8.49]), TEAS combined with opioids (RR *=* 2.39, 95% CI [1.81–3.15]), wrist–ankle acupuncture combined with opioids (RR *=* 2.83, 95% CI [1.00–7.98]), and fire needle combined with opioids (RR *=* 4.50, 95% CI [2.53–7.98]) were safer in the total incidence of adverse reactions and made the difference between groups statistically significant. Compared with TEAS combined with opioids, auricular acupuncture combined with opioids (RR *=* 1.92, 95% CI [1.24–2.97]) and electroacupuncture combined with opioids (RR *=* 1.93 95% CI [1.05–3.53]) were found to be safer in treatment of moderate to severe cancer pain (**Figure 3B**).

Based on SUCRA values, the ranking of nine interventions was as follows: auricular acupuncture combined with opioids (23.3%), electroacupuncture combined with opioids (25.1%), fire needle combined with opioids (27.2%), point embedding combined with opioids (42.6%), moxibustion combined with opioids (48.2%), body acupuncture combined with opioids (49.8%), wrist–ankle acupuncture combined with opioids (57.8%), TEAS combined with opioids (76.3%), and opioids alone (99.7%). Specific values are shown in **Figure 3C**.

#### 3.4.2 Incidence of nausea and vomiting

**Figure 4:**
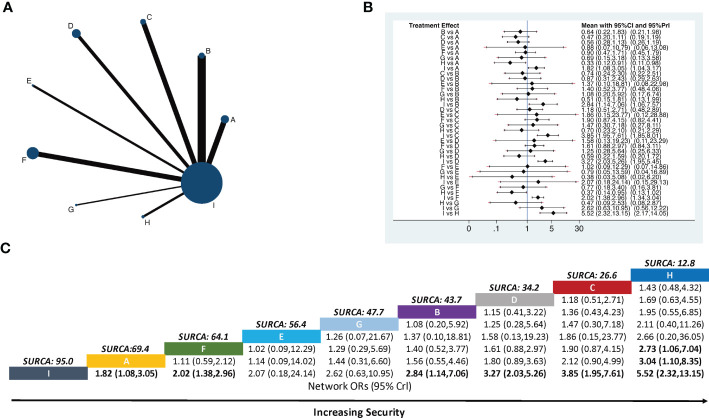
Network meta-analysis of the incidence of nausea and vomiting. **(A)** Network plot showing comparisons in the incidence of nausea and vomiting between each intervention. **(B)** Forest plot of the network meta-analysis comparing the incidence of nausea and vomiting in each treatment. **(C)** Schematic detailing the most secure treatments according to the surface under the cumulative ranking curve analysis (SUCRA). **A,** Body acupuncture combined with opioids; **B,** moxibustion combined with opioids; **C,** electroacupuncture combined with opioids; **D,** auricular acupuncture combined with opioids; **E,** point embedding combined with opioids; **F,** TEAS combined with opioids; **G,** wrist–ankle acupuncture combined with opioids; **H,** fire needle combined with opioids; **(I)** opioids.

Nine therapies were described in 26 studies that included the number of patients with nausea and vomiting (**Figure 4A**). For nausea and vomiting incidence, the pairwise meta-analysis comparing each intervention against opioids revealed that fire needle combined with opioids (RR *=* 5.52, 95% CI [2.32–13.15]), electroacupuncture combined with opioids (RR *=* 3.85, 95% CI [1.95–7.61]), auricular acupuncture combined with opioids (RR *=* 3.27, 95% CI [2.03–5.26]), moxibustion combined with opioids (RR *=* 2.84, 95% CI [1.14–7.06]), TEAS combined with opioids (RR *=* 2.02, 95% CI [1.38–2.96]), and body acupuncture combined with opioids (RR *=* 1.82, 95% CI [1.08–3.05]) were significantly superior to opioids alone. Body acupuncture combined with opioids (RR *=* 3.04, 95% CI [1.10–8.35]) and TEAS combined with opioids (RR *=* 2.73, 95% CI [1.06–7.04]) had a higher incidence of nausea and vomiting when compared with fire needle combined with opioids (**Figure 4B**).

The ranking of nine interventions based on SUCRA values was as follows: fire needle combined with opioids (12.8%), electroacupuncture combined with opioids (26.6%), auricular acupuncture combined with opioids (34.2%), moxibustion combined with opioids (43.7%), wrist–ankle acupuncture combined with opioids (47.7%), point embedding combined with opioids (56.4%), TEAS combined with opioids (64.1%), body acupuncture combined with opioids (69.4%), and opioids alone (95.0%) (**Figure 4C**).

#### 3.4.3 Incidence of constipation

**Figure 5:**
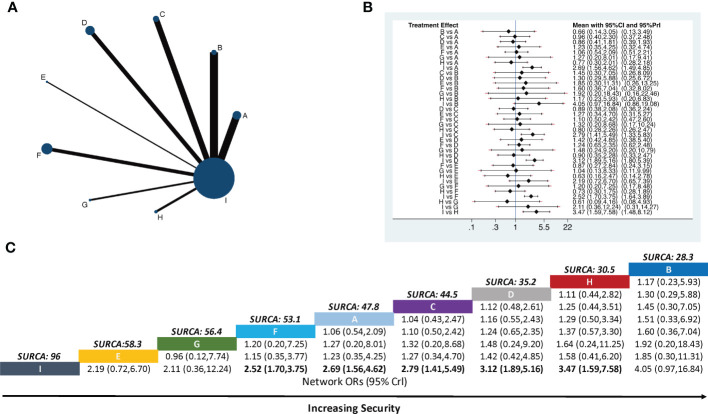
Network meta-analysis of the incidence of constipation. **(A)** Network plot showing comparisons in the incidence of constipation between each intervention. **(B)** Forest plot of the network meta-analysis comparing the incidence of constipation in each treatment. **(C)** Schematic detailing the most secure treatments according to the surface under the cumulative ranking curve analysis (SUCRA). **A,** Body acupuncture combined with opioids; **B,** moxibustion combined with opioids; **C,** electroacupuncture combined with opioids; **D,** auricular acupuncture combined with opioids; **E,** point embedding combined with opioids; **F,** TEAS combined with opioids; **G,** wrist–ankle acupuncture combined with opioids; **H,** fire needle combined with opioids; **I,** opioids.

In total, 25 studies reported the incidence of constipation, which constituted eight pairs of direct comparisons (no closed loop). The above results are detailed in **Figure 5A**. There were 25 pair comparisons in the NMA in terms of the incidence of constipation, and five indicated statistically significant differences. Fire needle combined with opioids (RR *=* 3.47, 95% CI [1.59–7.58]), auricular acupuncture combined with opioids (RR *=* 3.12, 95% CI [1.89–5.16]), electroacupuncture combined with opioids (RR *=* 3.04, 95% CI [1.53–6.08]), electroacupuncture combined with opioids (RR *=* 2.79, 95% CI [1.41–5.49]), body acupuncture combined with opioids (RR *=* 2.69, 95% CI [1.56–4.62]), and TEAS combined with opioids (RR *=* 2.52, 95% CI [1.70–3.75]) were significantly superior to opioids alone (**Figure 5B**).

The ranking of nine interventions based on SUCRA values was as follows: moxibustion combined with opioids (28.3%), fire needle combined with opioids (30.5%), auricular acupuncture combined with opioids (35.2%), electroacupuncture combined with opioids (44.5%), body acupuncture combined with opioids (47.8%), TEAS combined with opioids (53.1%), wrist–ankle acupuncture combined with opioids (56.4%), point embedding combined with opioids (58.3%), and opioids alone (96.00%) (**Figure 5C**).

### 3.5 Synthetic sorting bubble diagrams

**Figure 6:**
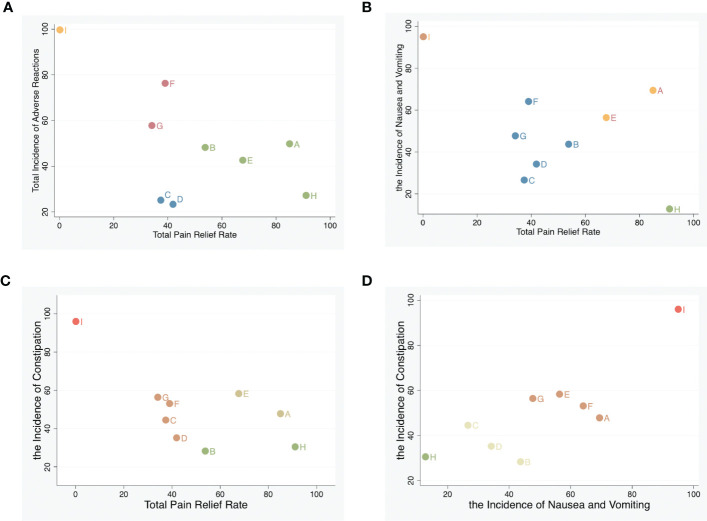
Synthetic sorting bubble diagram plot for outcomes. **(A)** Bubble diagram plot for the total pain relief rate and adverse reactions; **(B)** bubble diagram plot for total pain relief rate and nausea and vomiting. **(C)** Bubble diagram plot for total pain relief rate and constipation. **(D)** Bubble diagram plot for nausea and vomiting, and constipation. Note: Interventions with the same color belong to the same regimen, and interventions located in the lower left corner indicate optimal therapy for two different outcomes. **A,** Body acupuncture combined with opioids; **B,** moxibustion combined with opioids; **C,** electroacupuncture combined with opioids; **D,** auricular acupuncture combined with opioids; **E,** point embedding combined with opioids; **F,** TEAS combined with opioids; **G,** wrist–ankle acupuncture combined with opioids; **H,** fire needle combined with opioids; **I,** opioids.

We used synthetic sorting bubble diagrams to comprehensively present the relatively better intervention for cancer pain in this NMA. Bubble plots indicated that, considering the total pain relief rate and incidence of adverse reactions, fire needle combined with opioids was the preferred treatment. It has the highest pain relief rate and the lowest incidence of adverse reactions, especially nausea and vomiting. The second is body acupuncture combined with opioids, which has a high pain relief rate and a low incidence of constipation, but a high incidence of nausea and vomiting. Opioids alone has the lowest pain relief rate and the highest incidence of adverse reactions (nausea, vomiting, or constipation) (**Figure 6**).

### 3.6 Comparison-adjusted funnel plot

**Figure 7:**
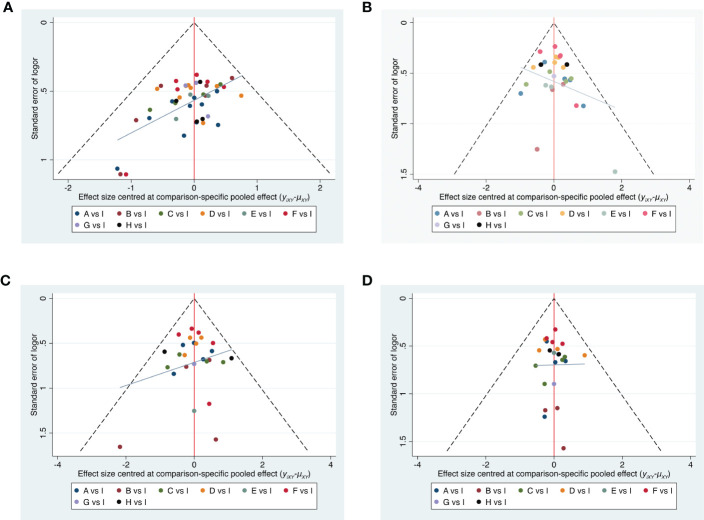
Funnel plots. **(A)** Total pain relief rate. **(B)** Total adverse reactions. **(C)** The incidence of Nausea and vomiting. **(D)** The incidence of constipation. **A**, Body acupuncture combined with opioids; **B**, moxibustion combined with opioids; **C**, electroacupuncture combined with opioids; **D**, auricular acupuncture combined with opioids; **E**, point embedding combined with opioids; **F**, TEAS combined with opioids; **G**, wrist–ankle acupuncture combined with opioids; **H**, fire needle combined with opioids; **I**, opioids.

Publication bias was detected *via* comparison-adjusted funnel plots for four outcomes, respectively. All included randomized controlled trials had an overall bias with some concerns, suggesting that there may be some publication bias or a small sample effect in the inclusion study (**Figure 7**).

## 4 Discussion

### 4.1 Interpretation of results and comparison with previous research

Acupuncture points can be stimulated with different methods, including invasive and noninvasive stimulation. It is widely used in the treatment of various pain conditions. During the thousands of years practicing in China, a wide range of technical manipulations conducted by needle, magnetic bead, fire, electricity, and even the operator’s fingers were applied to muscle or soft tissue at specific body locations to remotely regulate body function (68). Studies have pointed out that acupuncture inhibits both the sensory and the affective components of inflammatory pain, acting through peripheral, spinal, and supraspinal mechanisms with the involvement of a battery of bioactive molecules (7, 69, 70). Of these, opioids play a central role in pain (7).

In this study, NMA was used to compare the effectiveness and safety of APS combined with opioids in treating moderate and severe cancer pain. A stratified analysis was conducted on opioid-related adverse reactions (including nausea and vomiting). Finally, the quantitative sequencing results of different outcome indicators were integrated to find the best intervention measures for the treatment of moderate and severe cancer pain. The results showed that compared with opioids alone, eight APS therapies (body acupuncture, moxibustion, electroacupuncture, auricular acupuncture, point embedding, TEAS, wrist–ankle acupuncture, and fire needle) could improve the rate of pain relief and reduce the incidence of opioid-related adverse reactions. These findings were consistent with findings in previous studies and reviews (5, 71). Fire needle combined with opioids was considered to be the most effective treatment for moderate to severe cancer pain. Fire needle is a traditional form of acupuncture that combines conventional acupuncture and cauterization with heated needle therapy. Previous studies have found that the fire needle is widely employed to relieve acute and chronic pain (72–74). Studies showed that the fire needle could regulate Wnt/ERK multi-signal pathways bidirectionally, which have been shown to be closely associated with neuropathic pain (73).

In terms of the incidence of adverse reactions, auricular acupuncture had the lowest incidence of total adverse reactions. It is a complementary alternative therapy based on the theory that dysfunction in the body’s organs causes changes in various areas outside the ears by stimulating these response points, which were connected to the “pathological” organs, to improve the function of the organ and thus relieve pain. At present, a number of studies have shown that auricular acupuncture has a positive effect on the treatment of various pains with a lower incidence of adverse reactions (75, 76). The analgesic effects of auricular acupuncture may be explained by the stimulation of the auricular branch of the vagal nerve (77). Opioids commonly cause gastrointestinal adverse reactions such as nausea, vomiting, and constipation, restricting their dosage for chronic pain control (78). In terms of the incidence of nausea and vomiting, APS combined with opioids was lower than opioids alone, and fire needle combined with opioids was the lowest. There was good clinical evidence showing that acupuncture had some effects in preventing or attenuating nausea and vomiting (79). Recent research also suggested that APS may be a kind of alternative therapy for the prevention and treatment of tumor nausea and vomiting (80).In terms of the incidence of constipation, moxibustion combined with opioids had the lowest incidence of constipation. A recent meta-analysis showed that combined therapy with both medicine and acupuncture has insightful potential for future clinical cancer patient management of constipation problems.

Considering the total pain relief rate and the incidence of adverse reactions, fire needle, body acupuncture, point embedding, and moxibustion were considered to be effective and safe methods in treating moderate to severe cancer pain. The combination of fire needle and opioids had the highest rate of total pain relief, and a lower incidence of adverse reactions, especially vomiting. This would be considered to be a priority option. The total pain relief rate of body acupuncture combined with opioids was second to fire needle but had a higher incidence of adverse reactions among the eight therapies, especially nausea and vomiting, which needed to be balanced. Point embedding also performed well in pain relief and the consideration of safety. Current studies on point embedding mainly focus on metabolic-related diseases (such as obesity, diabetes, etc.), and there were few studies on pain management. Only three studies were included in this study, and the evidence quality was relatively unsatisfactory; therefore, the conclusions should be treated with caution. In addition, the comprehensive effect of the fire needle and moxibustion was better. They both belong to traditional Chinese medicine’s warm and hot therapies that could dredge meridian to relieve pain. This might be why they were superior to other therapies.

### 4.2 Strengths and limitations of this study

We performed a comprehensive examination of the efficacy and safety of eight APS therapies in patients with moderate to severe cancer pain. The outcome measures were determined for the total pain relief rate and the incidence of adverse reactions. Given the large sample size and narrower confidence intervals applied in this network meta-analysis, we believed that the findings were reliable. This review has some limitations. Firstly, the heterogeneity of the baseline characteristics, such as male-to-female ratio, sample size, site, and duration were not analyzed. Second, the sample sizes were relatively small, which may reduce questions about the applicability and accuracy of the results. Thirdly, most studies were based on short follow-up periods, which limited long-term effects. Lastly, some outcomes, such as analgesic dose and quality of life, were not analyzed because of limited data.

## 5 Conclusion

Eight methods included in the present network analysis had different advantages in treating moderate to severe cancer pain. Based on the results of the network meta-analysis and probability ranking analysis, fire needle combined with opioids, filiform needle combined with opioids, moxibustion combined with opioids, and point embedding combined with opioids might be the four best ways to treat moderate to severe cancer pain. Of course, clinicians should thoroughly evaluate the degree of pain and the occurrence of adverse reactions and then make individualized treatment plans for patients to improve the analgesic effect. In addition, this network meta-analysis is vital for future research, highlighting the need for adequately designed RCTs and more head-to-head comparisons of the most commonly used dressings in this field. Currently, there is scant evidence, mainly indirect and mostly from small trials, with a risk of unclear bias.

## Data availability statement

The original contributions presented in the study are included in the article/**Supplementary Material**. Further inquiries can be directed to the corresponding author.

## Author contributions

QZ: conceptualization, methodology, formal analysis, and writing the original draft. LF: writing—review and editing and supervision. YW: conceptualization, validation, and data curation. YC: methodology and formal analysis. YY: writing review and editing and supervision. MZ: conceptualization, Project administration, and supervision. All authors contributed to the article and approved the submitted version.
