# Supplementary material for: Efficacy and safety of acupuncture-point stimulation combined with opioids for the treatment of moderate to severe cancer pain: a network meta-analysis of randomized controlled trials
Source: Front Oncol. 2023 Jun 2;13:1166580. doi: 10.3389/fonc.2023.1166580 (PMC10272816; doi:10.3389/fonc.2023.1166580)
Supplement: Supplementary file 1 [file DataSheet_1.docx]

Supplementary Material

**Efficacy and Safety of Acupuncture-Point Stimulation Combined with Opioids for Treatment of Moderate to Severe Cancer Pain: A Network Meta-Analysis of Randomized Controlled Trials**

Qinglin Zhang1,2 Yuntong Yuan1 , Meiling Zhang2 , Ying Wang1,3, Yiyuan Cui3, Li Feng3,*

Qinglin Zhang^1,2^ Yuntong Yuan^1^ , Meiling Zhang^2^ , Ying Wang^1,3^, Yiyuan Cui^3^, Li Feng^3,*^

**Authors’ affiliations:**

^1^ Dongzhimen Hospital, Beijing University of Chinese Medicine, Beijing, China

^2^ The Third Clinical Medical College of Zhejiang Chinese Medical University, Zhejiang Province, China

^3^ Cancer Hospital Chinese Academy of Medical Sciences, Beijing, China

*** Correspondence:** *Li Feng, Cancer Hospital Chinese Academy of Medical Sciences，17 Panjiayuan Nanli, Chaoyang District, Beijing, China.

**Email:** [fengli663@126.com](mailto:fengli663@126.com)

# Supplementary Data

**Data 1 The search strategy for each database**

*PubMed search strategy*

**Search Date: June 30, 2022**

**----------------------------------------------------------------------------------------------------------------------**

#1 Cancer Pain [Mesh]

#2 ((((((((((((((((((((((((((((((((((((((((Cancer Pains[Title/Abstract]) OR (Pain, Cancer[Title/Abstract])) OR (Pains, Cancer[Title/Abstract])) OR (Cancer-Associated Pain[Title/Abstract])) OR (Cancer Associated Pain[Title/Abstract])) OR (Cancer-Associated Pains[Title/Abstract])) OR (Pain, Cancer-Associated[Title/Abstract])) OR (Pains, Cancer-Associated[Title/Abstract])) OR (Neoplasm-Related Pain[Title/Abstract])) OR (Neoplasm Related Pain[Title/Abstract])) OR (Neoplasm-Related Pains[Title/Abstract])) OR (Pain, Neoplasm-Related[Title/Abstract])) OR (Pains, Neoplasm-Related[Title/Abstract])) OR (Pains, Neoplasm-Related Oncological Pain[Title/Abstract])) OR (Oncological Pains[Title/Abstract])) OR (Pain, Oncological[Title/Abstract])) OR (Pains, Oncological[Title/Abstract])) OR (Tumor-Related Pain[Title/Abstract])) OR (Pain, Tumor-Related[Title/Abstract])) OR (Pains, Tumor-Related[Title/Abstract])) OR (Tumor Related Pain[Title/Abstract])) OR (Tumor-Related Pains[Title/Abstract])) OR (Tumor-Associated Pain[Title/Abstract])) OR (Pain, Tumor-Associated[Title/Abstract])) OR (Pains, Tumor-Associated[Title/Abstract])) OR (Tumor Associated Pain[Title/Abstract])) OR (Tumor-Associated Pains[Title/Abstract])) OR (Oncology Pain[Title/Abstract])) OR (Oncology Pains[Title/Abstract])) OR (Pain, Oncology[Title/Abstract])) OR (Pains, Oncology[Title/Abstract])) OR (Cancer-Related Pain[Title/Abstract])) OR (Cancer Related Pain[Title/Abstract])) OR (Cancer-Related Pains[Title/Abstract])) OR (Pain, Cancer-Related[Title/Abstract])) OR (Pains, Cancer-Related[Title/Abstract])) OR (Neoplasm-Associated Pain[Title/Abstract])) OR (Neoplasm Associated Pain[Title/Abstract])) OR (Neoplasm-Associated Pains[Title/Abstract])) OR (Pain, Neoplasm-Associated[Title/Abstract])) OR (Pains, Neoplasm-Associated[Title/Abstract])

#3 #1 OR #2

#4 (((((((Acupoint[MeSH Terms]) OR (Acupuncture[MeSH Terms])) OR (Acupressure[MeSH Terms])) OR (Acupuncture, Ear[MeSH Terms])) OR (Acupuncture Points[MeSH Terms])) OR (Acupuncture Analgesia[MeSH Terms])) OR (Massage[MeSH Terms])) OR (Moxibustion[MeSH Terms])

#5 ((((((((((((((((((((((((((((((((((((((((Acupuncture Point[Title/Abstract]) OR (Point, Acupuncture[Title/Abstract])) OR (Points, Acupuncture[Title/Abstract])) OR (Acupoints[Title/Abstract])) OR (Acupoint[Title/Abstract])) OR (Pharmacopuncture[Title/Abstract])) OR (Shiatsu[Title/Abstract])) OR (Zhi Ya[Title/Abstract])) OR (Chih Ya[Title/Abstract])) OR (Shiatzu[Title/Abstract])) OR (Catgut Embedment in Acupoint Therapy[Title/Abstract])) OR (Acupunctures, Ear[Title/Abstract])) OR (Ear Acupunctures[Title/Abstract])) OR (Auricular Acupuncture[Title/Abstract])) OR (Ear Acupuncture[Title/Abstract])) OR (Acupuncture, Auricular[Title/Abstract])) OR (Acupunctures, Auricular[Title/Abstract])) OR (Auricular Acupunctures[Title/Abstract])) OR (Acupuncture Point[Title/Abstract])) OR (Point, Acupuncture[Title/Abstract])) OR (Points, Acupuncture[Title/Abstract])) OR (Acupoints[Title/Abstract])) OR (Acupoint[Title/Abstract])) OR (Acupoint Pressure Therapy[Title/Abstract])) OR (Analgesia, Acupuncture[Title/Abstract])) OR (Acupuncture Anesthesia[Title/Abstract])) OR (Anesthesia, Acupuncture[Title/Abstract])) OR (Points,Scalp Stimulation Areas[Title/Abstract])) OR (Acupoint Iontophoresis Therapy[Title/Abstract])) OR (Zone Therapy[Title/Abstract])) OR (Therapies, Zone[Title/Abstract])) OR (Zone Therapies[Title/Abstract])) OR (Therapy, Zone[Title/Abstract])) OR (Massage Therapy[Title/Abstract])) OR (Massage Therapies[Title/Abstract])) OR (Therapies, Massage[Title/Abstract])) OR (Therapy, Massage[Title/Abstract])) OR (Electroacupuncture[Title/Abstract])) OR (Fire Needle Therapy[Title/Abstract])) OR (Moxibustion[Title/Abstract])) OR (Moxabustion[Title/Abstract])

#6 #4 OR #5

#7 #3 AND #6

*EMBASE search strategy*

**Search Date: June 30, 2022**

**----------------------------------------------------------------------------------------------------------------------**

('cancer pain'/exp OR 'cancer pains':ti,ab,kw OR 'pain, cancer':ti,ab,kw OR 'pains, cancer':ti,ab,kw OR 'cancer-associated pain':ti,ab,kw OR 'cancer associated pain':ti,ab,kw OR 'cancer-associated pains':ti,ab,kw OR 'pain, cancer-associated':ti,ab,kw OR 'pains, cancer-associated':ti,ab,kw OR 'neoplasm-related pain':ti,ab,kw OR 'neoplasm related pain':ti,ab,kw OR 'neoplasm-related pains':ti,ab,kw OR 'pain, neoplasm-related':ti,ab,kw OR 'pains, neoplasm-related':ti,ab,kw OR 'oncological pain':ti,ab,kw OR 'oncological pains':ti,ab,kw OR 'pain, oncological':ti,ab,kw OR 'pains, oncological':ti,ab,kw OR 'tumor-related pain':ti,ab,kw OR 'pain, tumor-related':ti,ab,kw OR 'pains, tumor-related':ti,ab,kw OR 'tumor related pain':ti,ab,kw OR 'tumor-related pains':ti,ab,kw OR 'tumor-associated pain':ti,ab,kw OR 'pain, tumor-associated':ti,ab,kw OR 'pains, tumor-associated':ti,ab,kw OR 'tumor associated pain':ti,ab,kw OR 'tumor-associated pains':ti,ab,kw OR 'oncology pain':ti,ab,kw OR 'oncology pains':ti,ab,kw OR 'pain, oncology':ti,ab,kw OR 'pains, oncology':ti,ab,kw OR 'cancer-related pain':ti,ab,kw OR 'cancer related pain':ti,ab,kw OR 'cancer-related pains':ti,ab,kw OR 'pain, cancer-related':ti,ab,kw OR 'pains, cancer-related':ti,ab,kw OR 'neoplasm-associated pain':ti,ab,kw OR 'neoplasm associated pain':ti,ab,kw OR 'neoplasm-associated pains':ti,ab,kw OR 'pain, neoplasm-associated':ti,ab,kw OR 'pains, neoplasm-associated':ti,ab,kw) AND ('electroacupuncture':ab,kw,ti OR 'acupuncture':ti,ab,kw OR 'acupuncture treatment':ti,ab,kw OR 'acupuncture treatments':ti,ab,kw OR 'treatment, acupuncture':ti,ab,kw OR 'therapy, acupuncture':ti,ab,kw OR 'pharmacoacupuncture treatment':ti,ab,kw OR 'treatment, pharmacoacupuncture':ti,ab,kw OR 'pharmacoacupuncture therapy':ti,ab,kw OR 'therapy, pharmacoacupuncture':ti,ab,kw OR 'acupotomy':ti,ab,kw OR 'acupotomies':ti,ab,kw OR 'acupunctures, ear':ti,ab,kw OR 'ear acupunctures':ti,ab,kw OR 'auricular acupuncture':ti,ab,kw OR 'ear acupuncture':ti,ab,kw OR 'acupuncture, auricular':ti,ab,kw OR 'acupunctures, auricular':ti,ab,kw OR 'auricular acupunctures':ti,ab,kw OR 'acupuncture point':ti,ab,kw OR 'point, acupuncture':ti,ab,kw OR 'points, acupuncture':ti,ab,kw OR 'acupoints':ti,ab,kw OR 'acupoint':ti,ab,kw OR 'zone therapy':ti,ab,kw OR 'therapies, zone':ti,ab,kw OR 'zone therapies':ti,ab,kw OR 'therapy, zone':ti,ab,kw OR 'massage therapy':ti,ab,kw OR 'massage therapies':ti,ab,kw OR 'therapies, massage':ti,ab,kw OR 'acupoint patching':ti,ab,kw OR 'filiform needle':ti,ab,kw OR 'fire needle':ti,ab,kw OR 'moxibustion':ti,ab,kw OR 'moxabustion':ti,ab,kw)

*Web of Science*

**Search Date: June 30, 2022**

**----------------------------------------------------------------------------------------------------------------------**

TS=( 'cancer pain*’ OR 'cancer pains*’ OR 'pain, cancer*’ OR 'pains, cancer*’ OR 'cancer-associated pain*’ OR 'cancer associated pain*’ OR 'cancer-associated pains*’ OR 'pain, cancer-associated*’ OR 'pains, cancer-associated*’ OR 'neoplasm-related pain*’ OR 'neoplasm related pain*’ OR 'neoplasm-related pains*’ OR 'pain, neoplasm-related*’ OR 'pains, neoplasm-related*’ OR 'oncological pain*’ OR 'oncological pains*’ OR 'pain, oncological*’ OR 'pains, oncological*’ OR 'tumor-related pain*’ OR 'pain, tumor-related*’ OR 'pains, tumor-related*’ OR 'tumor related pain*’ OR 'tumor-related pains*’ OR 'tumor-associated pain*’ OR 'pain, tumor-associated*’ OR 'pains, tumor-associated*’ OR 'tumor associated pain*’ OR 'tumor-associated pains*’ OR 'oncology pain*’ OR 'oncology pains*’ OR 'pain, oncology*’ OR 'pains, oncology*’ OR 'cancer-related pain*’ OR 'cancer related pain*’ OR 'cancer-related pains*’ OR 'pain, cancer-related*’ OR 'pains, cancer-related*’ OR 'neoplasm-associated pain*’ OR 'neoplasm associated pain*’ OR 'neoplasm-associated pains*’ OR 'pain, neoplasm-associated*’ OR 'pains, neoplasm-associated*’) AND TS=('electroacupuncture':ab,kw,ti OR 'acupuncture*’ OR 'acupuncture treatment*’ OR 'acupuncture treatments*’ OR 'treatment, acupuncture*’ OR 'therapy, acupuncture*’ OR 'pharmacoacupuncture treatment*’ OR 'treatment, pharmacoacupuncture*’ OR 'pharmacoacupuncture therapy*’ OR 'therapy, pharmacoacupuncture*’ OR 'acupotomy*’ OR 'acupotomies*’ OR 'acupunctures, ear*’ OR 'ear acupunctures*’ OR 'auricular acupuncture*’ OR 'ear acupuncture*’ OR 'acupuncture, auricular*’ OR 'acupunctures, auricular*’ OR 'auricular acupunctures*’ OR 'acupuncture point*’ OR 'point, acupuncture*’ OR 'points, acupuncture*’ OR 'acupoints*’ OR 'acupoint*’ OR 'zone therapy*’ OR 'therapies, zone*’ OR 'zone therapies*’ OR 'therapy, zone*’ OR 'massage therapy*’ OR 'massage therapies*’ OR 'therapies, massage*’ OR 'acupoint patching*’ OR 'filiform needle*’ OR 'fire needle*’ OR 'moxibustion*’ OR 'moxabustion*’) AND TS=(‘randomised controlled trial’OR ‘random*’ OR ‘controlled clinical trial’ OR ‘rct’)

*The Cochrane Library*

**Search Date: June 30, 2022**

**----------------------------------------------------------------------------------------------------------------------**

#1 Mesh descriptor: [cancer pain] explode all trees;

#2 'cancer pains':ti,ab,kw OR 'pain, cancer':ti,ab,kw OR 'pains, cancer':ti,ab,kw OR 'cancer-associated pain':ti,ab,kw OR 'cancer associated pain':ti,ab,kw OR 'cancer-associated pains':ti,ab,kw OR 'pain, cancer-associated':ti,ab,kw OR 'pains, cancer-associated':ti,ab,kw OR 'neoplasm-related pain':ti,ab,kw OR 'neoplasm related pain':ti,ab,kw OR 'neoplasm-related pains':ti,ab,kw OR 'pain, neoplasm-related':ti,ab,kw OR 'pains, neoplasm-related':ti,ab,kw OR 'oncological pain':ti,ab,kw OR 'oncological pains':ti,ab,kw OR 'pain, oncological':ti,ab,kw OR 'pains, oncological':ti,ab,kw OR 'tumor-related pain':ti,ab,kw OR 'pain, tumor-related':ti,ab,kw OR 'pains, tumor-related':ti,ab,kw OR 'tumor related pain':ti,ab,kw OR 'tumor-related pains':ti,ab,kw OR 'tumor-associated pain':ti,ab,kw OR 'pain, tumor-associated':ti,ab,kw OR 'pains, tumor-associated':ti,ab,kw OR 'tumor associated pain':ti,ab,kw OR 'tumor-associated pains':ti,ab,kw OR 'oncology pain':ti,ab,kw OR 'oncology pains':ti,ab,kw OR 'pain, oncology':ti,ab,kw OR 'pains, oncology':ti,ab,kw OR 'cancer-related pain':ti,ab,kw OR 'cancer related pain':ti,ab,kw OR 'cancer-related pains':ti,ab,kw OR 'pain, cancer-related':ti,ab,kw OR 'pains, cancer-related':ti,ab,kw OR 'neoplasm-associated pain':ti,ab,kw OR 'neoplasm associated pain':ti,ab,kw OR 'neoplasm-associated pains':ti,ab,kw OR 'pain, neoplasm-associated':ti,ab,kw OR 'pains, neoplasm-associated':ti,ab,kw)；

#3 #1 OR #2

#4 Mesh descriptor: [acupuncture] explode all trees;

#5 Mesh descriptor: [electroacupuncture] explode all trees;

#6 Mesh descriptor: [auricular acupuncture] explode all trees;

#7 Mesh descriptor: [acupuncture therapy] explode all trees;

#8 Mesh descriptor: [moxibustion] explode all trees;

#9 Mesh descriptor: [acupuncture analgesia] explode all trees;

#10 Mesh descriptor: [acupressure] explode all trees;

#11 'acupuncture treatment':ti,ab,kw OR 'acupuncture treatments':ti,ab,kw OR 'treatment, acupuncture':ti,ab,kw OR 'therapy, acupuncture':ti,ab,kw OR 'pharmacoacupuncture treatment':ti,ab,kw OR 'treatment, pharmacoacupuncture':ti,ab,kw OR 'pharmacoacupuncture therapy':ti,ab,kw OR 'therapy, pharmacoacupuncture':ti,ab,kw OR 'acupotomy':ti,ab,kw OR 'acupotomies':ti,ab,kw OR 'acupunctures, ear':ti,ab,kw OR 'ear acupunctures':ti,ab,kw OR 'ear acupuncture':ti,ab,kw OR 'acupuncture, auricular':ti,ab,kw OR 'acupunctures, auricular':ti,ab,kw OR 'auricular acupunctures':ti,ab,kw OR 'acupuncture point':ti,ab,kw OR 'point, acupuncture':ti,ab,kw OR 'points, acupuncture':ti,ab,kw OR 'acupoints':ti,ab,kw OR 'acupoint':ti,ab,kw OR 'zone therapy':ti,ab,kw OR 'therapies, zone':ti,ab,kw OR 'zone therapies':ti,ab,kw OR 'therapy, zone':ti,ab,kw OR 'massage therapy':ti,ab,kw OR 'massage therapies':ti,ab,kw OR 'therapies, massage':ti,ab,kw OR 'acupoint patching':ti,ab,kw OR 'filiform needle':ti,ab,kw OR 'fire needle':ti,ab,kw OR 'moxabustion':ti,ab,kw)

#12 #4 OR #5 OR #6 OR #7 OR #8 OR #9 OR #10 OR #11

#13 Mesh descriptor: [Randomized Controlled Trials as Topic] explode all trees;

#14 "Randomized Controlled Trials":ti,ab,kw or "random*":ti,ab,kw or "controlled clinical trial":ti,ab,kw or "rct":ti,ab,kw (Word variations have been searched);

#15 #13 OR #14

#14 #3 and #12 and #15

*CKNI search strategy*

**Search Date: June 30, 2022**

**----------------------------------------------------------------------------------------------------------------------**

(主题:( 癌性疼痛+瘤性疼痛+肿瘤相关性疼痛+肿瘤疼痛+癌痛+瘤相关疼痛+癌症疼痛) AND 主题:(镇痛+止痛+疼痛)AND 主题:(针灸疗法+针刺+灸法+针刺镇痛+针刺麻醉+经皮神经电刺激+穴位疗法+穴位注射) AND 摘要:(随机+随机分配+随机对照试验)

*CBM search strategy*

**Search Date: June 30, 2022**

**----------------------------------------------------------------------------------------------------------------------**

(（TKA=“癌痛” [不加权:扩展] + “癌性疼痛” [不加权:扩展] + “瘤性疼痛” [不加权:扩展] + “肿瘤相关性疼痛” [不加权:扩展] + “肿瘤疼痛” [不加权:扩展] + “瘤相关疼痛” [不加权:扩展] + “癌症疼痛” [不加权:扩展] ）AND（TKA=“针灸” [不加权:扩展] + “针刺” [不加权:扩展] + “火针” [不加权:扩展] + “电针” [不加权:扩展] + “毫针” [不加权:扩展] + “耳针” [不加权:扩展] + “耳穴” [不加权:扩展] + “磁珠” [不加权:扩展] + “耳豆” [不加权:扩展] + “穴位” [不加权:扩展] + “敷” [不加权:扩展] + “贴” [不加权:扩展] + “推拿” [不加权:扩展] + “按摩” [不加权:扩展] + “灸” [不加权:扩展] + “艾灸” [不加权:扩展]）AND ("随机对照试验"[不加权:扩展] OR "随机分配"[不加权:扩展] OR "随机"[摘要:智能])）

*VIP search strategy*

**Search Date: June 30, 2022**

**----------------------------------------------------------------------------------------------------------------------**

题名或关键词=（癌痛 OR 癌性疼痛 OR 瘤性疼痛 OR 肿瘤相关性疼痛 OR 肿瘤疼痛 OR 瘤相关疼痛 OR 癌症疼痛） AND题名或关键词=（针灸 OR 针刺 OR 火针 OR 电针 OR 毫针 OR 耳针 OR 耳穴 OR 耳豆 OR 敷 OR 贴 OR 推拿 OR 按摩 OR 艾灸 OR 灸 OR 穴位）AND文摘:(随机+随机分配+随机对照试验)

*Wanfang Database search strategy*

**Search Date: June 30, 2022**

**----------------------------------------------------------------------------------------------------------------------**

题名或关键词=（癌痛 OR 癌性疼痛 OR 瘤性疼痛 OR 肿瘤相关性疼痛 OR 肿瘤疼痛 OR 瘤相关疼痛 OR 癌症疼痛） AND题名或关键词=（针灸 OR 针刺 OR 火针 OR 电针 OR 毫针 OR 耳针 OR 耳穴 OR 耳豆 OR 敷 OR 贴 OR 推拿 OR 按摩 OR 艾灸 OR 灸 OR 穴位）AND文摘:(随机+随机分配+随机对照试验)

**Data 2** References for included RCTs (Chinese and English)

1. Na, X., Fangfang, M., Peiyu, C., Qi, F., Yongme, X., Guowang, Y., 2022. Clinical observation acupuncture for regulating the mind and relieving pain in the treatment of moderate and severe cancer pain. 03 (31), 334-337+424.

薛娜,马芳芳,程培育,富琦,徐咏梅,杨国旺.调神止痛针刺法治疗中重度癌痛临床观察[J].现代中西医结合杂志,2022,31(03):334-337+424.

1. Zhuo, C., Hong-yu, X., Qi, C., 2021. Clinical Observation on Acupuncture plus Oxycodone Hydrochloride Sustained Release Tablets for Severe Cancer Pain Caused by Vertebral Metastasis. Shanghai Journal of Acupuncture and Moxibustion 40 (4), 411-415.

陈卓,肖宏宇,程祺.针刺联合盐酸羟考酮控释片治疗椎体转移所致重度癌性疼痛的临床观察[J].上海针灸杂志,2021,40(04):411-415.DOI:10.13460/j.issn.1005-0957.2021.04.0411.

1. Liqiong, L., Xiaoxiao, Z., Xiang, T., 2021. Effect of thunder fire moxibustion on moderate and severe cancer pain. Research of Integrated Traditional Chinese and Western Medicine 13 (04), 284-285+288.

吕丽琼,朱晓晓,唐香.雷火灸干预中重度癌性疼痛效果观察[J].中西医结合研究,2021,13(04):284-285+288.

1. Ping, X., wen, X., Youhui, Y., Lu, Z., Yarong, T., 2021. Effect of thunder fire moxibustion combined with opioid drugs on cancer pain. Laboratory Medicine and Clinic 18 (19), 2875-2877.

许萍,许雯,袁友辉,张璐,唐亚荣.雷火灸联合阿片类药物治疗癌性疼痛的疗效观察[J].检验医学与临床,2021,18(19):2875-2877.

1. Lu, D.-R., Xia, Y.-Q., Chen, F., Wang, N.-J., He, S.-Q., Wang, F., Zhu, S.-J., 2021. Effect of electrothermal acupuncture on moderate to severe cancer pain with yin-cold stagnation: a randomized controlled trial. Zhongguo Zhen jiu= Chinese Acupuncture & Moxibustion 41 (2), 121-124.

芦殿荣,夏玉卿,陈枫,王宁军,何生奇,王芳,朱世杰.电热针对阴寒凝滞型中重度癌性躯体疼痛的影响：随机对照研究[J].中国针灸,2021,41(02):121-124+126.DOI:10.13703/j.0255-2930.20190320-0010.

1. Zhi-ling, Y., Ya-bin, G., 2021. Clinical Observation of Electroacupuncture Combined with Opioids in the Treatment of Cancerous Pain. Journal of Oncology in Chinese Medicine 3 (03), 30-35.

杨之泠,龚亚斌.电针联合阿片类药物治疗癌性疼痛的临床疗效观察[J].中医肿瘤学杂志,2021,3(03):30-35.DOI:10.19811/j.cnki.ISSN2096-6628.2021.03.000.

1. Run, C., Ruifang, Z., Ping, F., Weixia, H., 2021. The effect of auricular plaster therapy combined with oxycodone hydrochloride sustained release tablets on the number of pain outbreak ，PPI score and KPS score in cancer pain patients. Medical Journal of West China 33 (11), 1683-1686.

陈润,曾睿芳,方平,黄伟霞.耳穴压豆联合盐酸羟考酮缓释片对癌性疼痛患者疼痛爆发次数及PPI与KPS评分的影响[J].西部医学,2021,33(11):1683-1686.

1. Guo-dong, Z., Zhi-hui, Z., Hong-fang, S., 2021. Clinical Study of Acupoint Catgut Embedding Therapy on Colorectal Cancer Pain. World Latest Medicine Information 21 (20), 9-10.

臧国栋,赵志辉,孙红芳. 穴位埋线疗法对结直肠癌癌性疼痛的临床研究[J]. 世界最新医学信息文摘（连续型电子期刊）,2021,21(20):9-10. DOI:10.3969/j.issn.1671-3141.2021.20.005.

1. Jian-chun, P., Hui-ying, H., Sheng-ling, Z., 2021. Nursing with traditional Chinese medicine combined with percutaneous electrical acupoint stimulation for advanced lung cancer complicated with bone metastasis related pain. Chinese Journal of Clinical Oncology and Rehabilitation 28 (10), 1243-1246.

蒲建春,黄荟颖,郑生苓.中医护理干预联合经皮穴位电刺激对晚期肺癌伴骨转移疼痛患者的护理价值[J].中国肿瘤临床与康复,2021,28(10):1243-1246.

1. Ling-ling, W., Xue-dong, L., Bi-quan, Q., Ye-jing, L., Dan-qian, W., 2021. Efficacy Observation of Wrist-ankle Acupuncture Combined with Opioids for Primary Liver Cancer-related Pain. Shanghai Journal of Acupuncture and Moxibustion 40 (11), 1336-1340.

王玲玲,林雪冬,全碧泉,雷叶静,翁丹茜.腕踝针联合阿片类药物治疗肝癌癌痛的疗效观察[J].上海针灸杂志,2021,40(11):1336-1340.DOI:10.13460/j.issn.1005-0957.2021.11.1336.

1. Xiang-hong, W., Fei-hong, L., 2021. Study on clinical effect of wrist ankle acupuncture combined with oxycodone hydrochloride sustained-release tablets in the treatment of refractory cancer pain. Chinese Evidence-based Nursing 7 (18), 2497-2500.

韦湘红,梁飞红.腕踝针联合盐酸羟考酮缓释片治疗难治性癌痛的临床效果研究[J].循证护理,2021,7(18):2497-2500.

1. Jiao, G., Jie, C., Yan-hua, X., Chao, K., Mei-mei, H., Chen-guang, Y., 2020. Clinical effect of acupoint catgut-embedding combined with painkillers on bone metastatic cancer pain. Clinical Research and Practice 5 (07), 115-117.

缑娇,陈捷,谢燕华,康超,郝梅梅,杨晨光.穴位埋线联合止痛药治疗骨转移癌痛的临床效果[J].临床医学研究与实践,2020,5(07):115-117.DOI:10.19347/j.cnki.2096-1413.202007049.

1. Dan, L., Rui-Rui, S., Qing-Ling, L., Qiang, M., Yong-Lei, Z., Xue-Zhao, J., Jing, W., 2020. Acupuncture combined with opioid drugs on moderate and severe cancer pain: a randomized controlled trial. Zhongguo Zhen jiu= Chinese Acupuncture & Moxibustion 40 (3), 257-261.

李丹,孙瑞瑞,李庆羚,马强,曾永蕾,贾学昭,王茎.针刺联合阿片类药物治疗中重度癌性疼痛：随机对照研究[J].中国针灸,2020,40(03):257-261.DOI:10.13703/j.0255-2930.20190311-k0001.

1. Bing, L., Lin, L., Li, T., Cuiqing, D., Jinfang, J., 2020. Curative Effect and Mechanism of Heat-sensitive Moxibustion Combined with Western Medicine in Treating Severe Colon Cancer Pain of Deficient Healthy Qi and Blood Stasis Type. 44 (05), 539-543+549.

刘冰,李琳,童莉,丁翠青,吉金芳.热敏灸联合西药治疗正虚瘀结型结肠癌重度癌痛的疗效及作用机制探究[J].山东中医药大学学报,2020,44(05):539-543+549.DOI:10.16294/j.cnki.1007-659x.2020.05.017.

1. Jun, C., Haifa, Q., Jing, L., 2020. The clinic research of Back Shu point moxibustion plus oral oxycodone in cancer pain. Shaanxi Journal of Traditional Chinese Medicine 41 (01), 105-107.

陈军,乔海法,李静,刘奇,Karataye Va Kymba.艾灸背俞穴联合羟考酮缓释片干预癌痛临床研究[J].陕西中医,2020,41(01):105-107.

1. Li-xia, L., 2020. Therapeutic Efficacy of Heat-sensitive Moxibustion in Adjuvant Treatment of Moderate Liver Cancer Pain and Its Effects on TNF-α and IL-2. Shanghai Journal of Acupuncture and Moxibustion 39 (06), 692-696.

刘丽霞.热敏灸辅助治疗中度肝癌痛的疗效及对TNF-a、IL-2的影响[J].上海针灸杂志,2020,39(06):692-696.DOI:10.13460/j.issn.1005-0957.2020.06.0692.

1. Chu-ting;, X., Xin-wei, L., Yi-nuo, T., 2020. Clinical observation of 35 cases of lung cancer patients with pain treated by dense wave electroacupuncture combined with western medicine. Zhejiang Journal of Traditional Chinese Medicine 55 (04), 295-296.

徐楚婷,李新伟,谭伊诺.疏密波电针结合西药治疗肺癌患者疼痛35例疗效观察[J].浙江中医杂志,2020,55(04):295-296.DOI:10.13633/j.cnki.zjtcm.2020.04.035.

1. Chen, L.-x., Yan, F., 2020. Clinical study on auricular point sticking plus Western medicine for moderate gastric cancer pain. Journal of Acupuncture and Tuina Science 18 (4), 276-280.

陈丽霞, 闫峰. 耳穴贴压联合西药治疗中度胃癌疼痛的临床研究[J]. 针灸推拿医学（英文版）,2020,18(4):276-280.

1. Xin, G., Shi-Nian, Z., 2020. Clinical observation of filiform fire needling on moderate and severe pain in advanced cancer. Zhongguo Zhen jiu= Chinese Acupuncture & Moxibustion 40 (6), 601-604.

高新,张仕年.毫火针治疗癌症晚期中、重度疼痛临床观察[J].中国针灸,2020,40(06):601-604.DOI:10.13703/j.0255-2930.20190531-k0001.

1. Zhi-peng, Z., Yu-chun, N., 2020. Effect of oxycontin combined with fire needling in treating cancer pain. World Journal of Integrated Traditional and Western Medicine 15 (04), 753-756.

张志鹏,倪育淳.火针配合奥施康定治疗癌痛临床疗效观察[J].世界中西医结合杂志,2020,15(04):753-756.DOI:10.13935/j.cnki.sjzx.200442.

1. Chao, Z., Xiufang, W., Yinyin, H., Yulong, C., Liang, Z., Deng, L., 2019. Effect of acupuncture and moxibustion on bone metastasis cancer pain in community health service. Women's Health Research 2019 (20), 119-120.

张超,吴秀芳,惠银银,陈钰龙,左光亮,刘登.社区卫生服务中针灸治疗骨转移癌痛的效果[J].中外女性健康研究,2019(20):119-120.

1. Zhiwei, Q., 2019. Clinical study of acupuncture combined with opioids in the treatment of cancer pain. Modern Digestion & Intervention 24 (A01), 0310-0311.

钱志伟. 针刺联合阿片类药物治疗癌性疼痛的临床研究[J]. 现代消化及介入诊疗,2019(A01):0310-0311.

1. Can, W., 2019. Clinical Study on Electroacupuncture Combined with Hydromorphone for Moderate and Severe Cancer Pain of Stagnation of Static Blood Type. New Chinese Medicine 51 (10), 242-244.

王灿.电针联合氢吗啡酮治疗瘀血停滞型中重度癌性疼痛临床研究[J].新中医,2019,51(10):242-244.DOI:10.13457/j.cnki.jncm.2019.10.069.

1. Xiaodan, H., Shi, C., 2019. Effect of auricular point pressing pill combined with morphine on patients with moderate and severe cancer pain in hospice care. Chinese Community Doctors 35 (24), 90.

贺晓丹,陈实.耳穴压丸联合吗啡治疗中重度癌痛临终关怀患者的疗效探讨[J].中国社区医师,2019,35(24):90.

1. Weijie, B., Enming, L., Cantu, F., Zekun, L., Zhiqiang, Z., Meizhen, X., Luzhen, L., 2019. Clinical study on the treatment of cancer pain based on the theory "Fire to smooth, generally no pain". Chinese Manipulation and Rehabilitation Medicine 10 (15), 14-17.

白伟杰,刘恩明,方灿途,罗泽坤,张志强,许美珍,李陆振.基于“火以畅达,通则不痛”理论毫火针刺治疗癌性疼痛的临床研究[J].按摩与康复医学,2019,10(15):14-17.

1. Dian-rong, L., Sheng-qi, H., Li, F., Dian-xiang, L., Xiao-fen, Y., Fang, W., Yin, G., 2018. Clinical research in the treatment of moderate and severe bone metastasis pain with acupuncture for tonifying kidney and eliminating stasis. World Journal of Integrated Traditional and Western Medicine 13 (01), 116-120.

芦殿荣,何生奇,冯利,芦殿香,袁晓玢,王芳,高音.针刺补肾祛瘀法治疗中重度骨转移癌痛的临床研究[J].世界中西医结合杂志,2018,13(01):116-120.DOI:10.13935/j.cnki.sjzx.180132.

1. Ying, H., 2018. Effect of acupuncture and moxibustion combined with three steps drugs on moderate and severe cancer pain. Inner Mongolia Journal of Traditional Chinese Medicine 37 (05), 58-59.

黄颖.针灸联合三阶梯药物治疗中重度癌痛疗效观察[J].内蒙古中医药,2018,37(05):58-59.DOI:10.16040/j.cnki.cn15-1101.2018.05.041.

1. Hongyan, X., 2018. Effect of thunder fire moxibustion on moderate to severe cancer pain. Cardiovascular Disease Electronic Journal of Integrated Traditional Chinese and Western Medicine 6 (09), 147+150.

辛红艳.雷火灸治疗中重度癌性疼痛的效果观察[J].中西医结合心血管病电子杂志,2018,6(09):147+150.DOI:10.16282/j.cnki.cn11-9336/r.2018.09.103.

1. Hui, W., Ying, W., Hong-ming, F., 2018. Thirty Cases of Patients with Lung Cancer Pain Treated with Electro-acupuncture Based on Syndrome Differentiation. Henan Traditional Chinese Medicine 38 (03), 454-457.

王辉,王颖,方红明.电针辨证治疗肺癌癌痛30例[J].河南中医,2018,38(03):454-457.DOI:10.16367/j.issn.1003-5028.2018.03.0120.

1. Guo-dong, Z., Zhi-hui, Z., 2018. Clinical study on the treatment of cancer pain with fire needle. China Health Care & Nutrition 28 (27), 36-37.

臧国栋,赵志辉. 火针围刺治疗癌痛的临床研究[J]. 中国保健营养,2018,28(27):36-37. DOI:10.3969/j.issn.1004-7484.2018.27.021.

1. Fan, L., Gao, S., Wang, Y., Qi, Z., 2017. Clinical observation of acupuncture combined with western medicine in treatment of advanced lung cancer pain. Medical Journal of Chinese People’s Health 29 (11), 36-38.

范立勇,高世领,王玉强,齐增平.针刺联合西药治疗中晚期肺癌疼痛的临床观察[J].中国民康医学,2017,29(11):36-38.

1. Dehui, L., Chunxia, S., Huanfang, F., Xiao, W., Liying, W., 2017. Clinical study on acupuncture at Zusanli, Taichong and Hegu points combined with three-step analgesic ladder for treatment of gastric cancer pain. Journal of Guangzhou University of Traditional Chinese Medicine, 344-347.

李德辉,孙春霞,范焕芳,王骁,魏莉瑛.针刺足三里、太冲、合谷穴配合三阶梯止痛治疗胃癌痛临床观察[J].广州中医药大学学报,2017,34(03):344-347.

1. Jing, X., Zunhua, S., 2017. The Evaluation on the Curative Effect of Auricular Needle in Treating Hepatocellular Pain for 40 Cases. Chinese Medicine Modern Distance Education of China 15 (18), 112-113.

旋静,舒遵华.耳针治疗肝癌疼痛40例疗效评价[J].中国中医药现代远程教育,2017,15(18):112-113.

1. Yun, D., Xing-feng, Z., Juan-juan, P., Yu-lin, Y., 2017. The therapeutic efficacy of oxycodone hydrochloride prolonged release tablets combined with bioelectric stimulation in treatment of neuropatho-logical cancer pain. Journal of North Sichuan Medical College 32 (1), 30-32.

邓芸,卓兴峰,彭娟娟,杨玉玲.盐酸羟考酮缓释片联合生物电刺激对神经病理性癌痛的治疗效果[J].川北医学院学报,2017,32(01):30-32.

1. Qing-quan, W., Yu, C., Lei, Z., Ming, L., 2016. Treatment of 60 cases of cancer pain with auricular acupoint pressing bean combined with morphine sulfate sustained-release tablets. Journal of External Therapy of Traditional Chinese Medicine 25 (04), 18-19.

王庆全,陈豫,朱蕾,陆明.耳穴压豆联合硫酸吗啡缓释片治疗癌痛60例[J].中医外治杂志,2016,25(04):18-19.

1. Wei, S., Zi-li, Z., 2016. Clinical Observation of Auricular Press-needle Combined with Western Medicine Treating Cancer Pain. Chinese Manipulation and Rehabilitation Medicine 7 (09), 24-25.

孙唯,张子丽.耳穴揿针联合西药治疗癌性疼痛的临床观察[J].按摩与康复医学,2016,7(09):24-25.

1. Yougang, W., Changping, Z., 2016. Therapeutic effect of acupoint catgut embedding on cancer pain of lung cancer. Journal of Preventive Medicine of Chinese People's Liberation Army 34 (S1), 297-298.

魏有刚,周长萍.穴位埋线治疗肺癌癌性疼痛的疗效观察[J].解放军预防医学杂志,2016,34(S1):297-298.DOI:10.13704/j.cnki.jyyx.2016.s1.264.

1. Hui, Z., Wei-fang, H., Li-li, H., Li-hua, H., 2016. The influence of Traditional Chinese Medical Nursing Intervention Combined with Transcutaneous Electrical Acupoint Stimulation in the Treatment of Bone Metastasis Pain of Advanced Lung Cancer. Journal of Guizhou University of Traditional Chinese Medicine 38 (04), 85-89.

张慧,黄伟芳,侯黎莉,黄李华.中医护理干预联合经皮穴位电刺激对晚期肺癌伴骨转移疼痛患者的影响[J].贵阳中医学院学报,2016,38(04):85-89.DOI:10.16588/j.cnki.issn1002-1108.2016.04.023.

1. Yi, D., 2015. Effect of morphine sulfate controlled-release tablets combined with acupuncture on improving quality of life in elderly patients with cancer pain. Modern Journal of Integrated Traditional Chinese and Western Medicine 24 (06), 606-608.

邓懿.硫酸吗啡控释片联合针刺改善老年癌性疼痛患者生存质量的效果[J].现代中西医结合杂志,2015,24(06):606-608.

1. Qinggang, D., 2015. Clinical Study of Acupuncture at Mingmen and Guanyuan Acupionts Combined with Analgesic on the Treatment of Lumbar Spinal Metastatic Carcinoma Pains. Chinese Medicine Modern Distance Education of China 13 (07), 65-66.

丁庆刚.针刺命门及关元穴配合镇痛药治疗腰椎转移癌疼痛30例[J].中国中医药现代远程教育,2015,13(07):65-66.

1. Qiuhong, L., Wei, Z., Zhiqun, L., 2015. Curative Effect and Costs of Oxycontin Combined with Transcutaneous Electrical Nerve Stimulationn Treatment of Moderate or Severe Cancer Pain. The Practical Journal of Cancer 30 (06), 922-924.

李秋宏,郑伟,刘志群.盐酸羟考酮缓释片联合生物电刺激治疗中重度癌痛病患的效果和医疗费用研究[J].实用癌症杂志,2015,30(06):922-924.

1. Ming-hua, W., You-jie, Y., 2015. Efficacy of oxycodone hydrochloride sustained release tablets combined with bioelectrical stimulation in the treatment of moderate to severe cancer pain. Health Research 35 (05), 562-563,565.

翁明华,余友杰.盐酸羟考酮缓释片联合生物电刺激治疗中重度癌痛的疗效观察[J].健康研究,2015,35(05):562-563+565.

1. Qiaotong, H., Lian, C., Yunfeng, J., 2014. Effect of thunder fire moxibustion on moderate to severe cancer pain. Guangxi Journal of Traditional Chinese Medicine 37 (06), 37-38.

黄乔统,陈莲,蒋云峰.加用雷火灸干预治疗中重度癌性疼痛的效果观察[J].广西中医药,2014,37(06):37-38.

1. DING, J., HUANG, J., 2014. Curative effects and costs of oxycontin combined with bio-electric stimulation therapy in treatment of moderate or severe cancer pain. Chinese General Practice 17 (3), 325-327.

李俊,丁纪元,黄静.盐酸羟考酮缓释片联合生物电刺激治疗中重度癌痛的疗效及治疗费用分析[J].中国全科医学,2014,17(03):325-327.

1. HU ZW, H.L., 2012. Clinical observation on auricular acupressure therapy combined with strong opioids for moderate to severe cancer pain. Journal of Traditional Chinese Medicine 53 (13), 1123-1125.

钟敏钰,胡作为,黄琳,但丹,成薇婷.耳穴贴压联合强阿片类药物治疗中、重度癌痛50例疗效观察[J].中医杂志,2012,53(13):1123-1125.DOI:10.13288/j.11-2166/r.2012.13.029.

1. Weiji, L., Jinyuan, Y., 2012. Therapeutic effect of acupuncture and morphine in the treatment of advanced lung cancer pain. China Health Care & Nutrition 2012 (09), 78.

刘伟基,要金元. 针刺加吗啡治疗肺癌晚期疼痛疗效观察[J]. 中国保健营养（中旬刊）,2012(9):78-78.

1. Fengying, Y., Ailing, P., 2011. Clinical observation of auricular acupuncture combined with morphine sustained-release tablets in the treatment of cancer pain. Chinese Practical Journal of Rural Doctor 18 (2), 56-57.

杨凤英,彭爱玲. 耳针结合吗啡缓释片治疗癌痛的临床观察[J]. 中国实用乡村医生杂志,2011,18(2):56-57. DOI:10.3969/j.issn.1672-7185.2011.02.036.

1. Su-e, S., 2000. Clinical observation on analgesic effect of wrist and ankle needle combined with drugs in cancer patients. Chinese Acupuncture & Moxibustion 2000 (03), 15-16.

沈素娥.腕踝针加药物对癌症患者止痛效果临床观察[J].中国针灸,2000(03):15-16.DOI:10.13703/j.0255-2930.2000.03.008.

# Supplementary Figures

## Supplementary Figures 1 Risk of bias in included studies


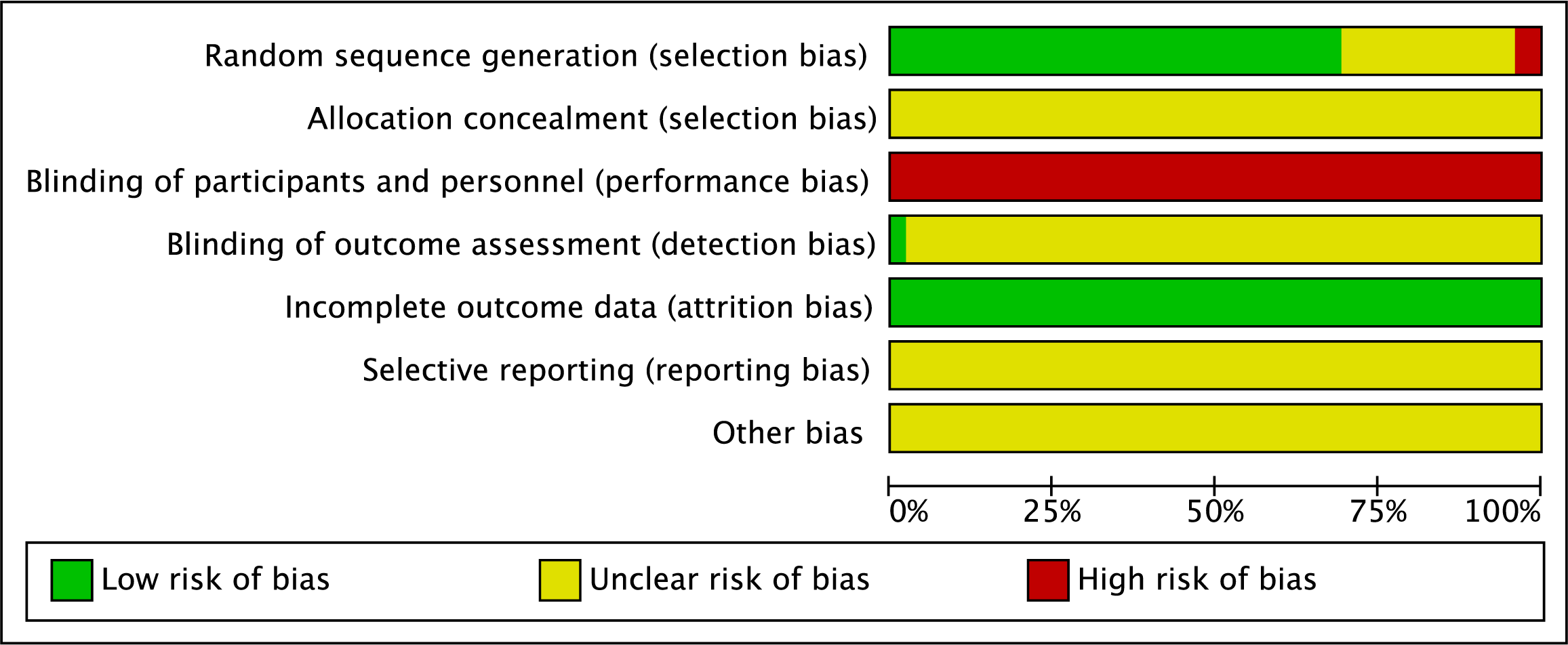


**Figures 1** Risk of bias in included studies

## Supplementary Figures 2 Review authors' judgements about each risk of bias item across all included studies


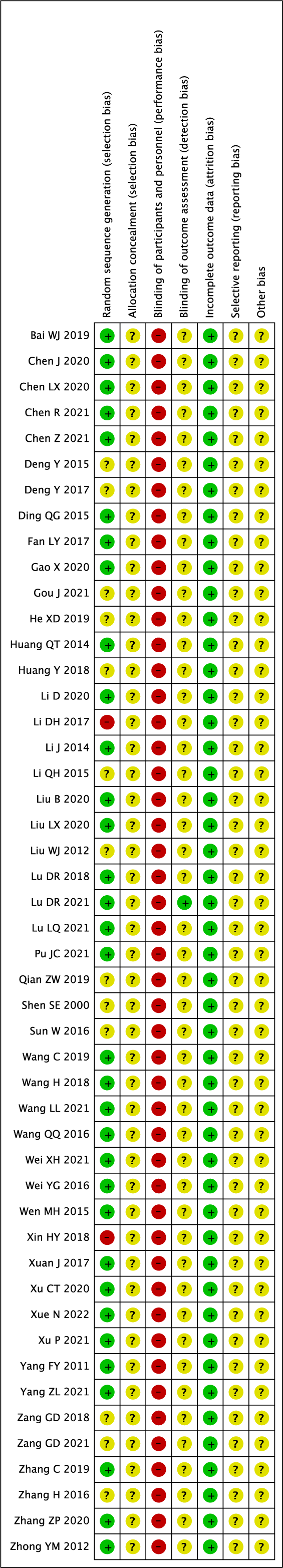


**Figures 2** Review authors' judgements about each risk of bias item across all included studies
