# Supplementary figures and images for: Efficacy and safety of acupuncture-point stimulation combined with opioids for the treatment of moderate to severe cancer pain: a network meta-analysis of randomized controlled trials
Source: Front Oncol. 2023 Jun 2;13:1166580. doi: 10.3389/fonc.2023.1166580 (PMC10272816; doi:10.3389/fonc.2023.1166580)

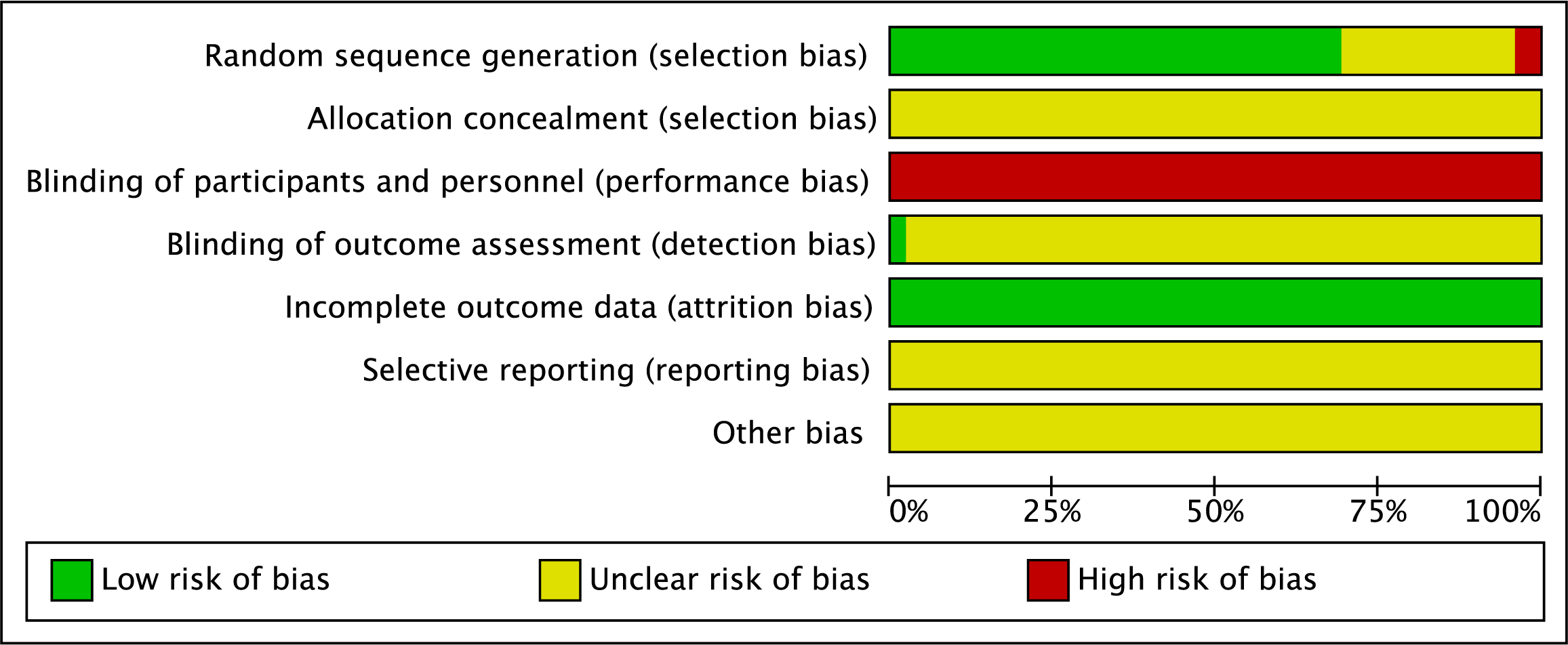

Supplement: Supplementary file 2 [file Image_1.jpeg]

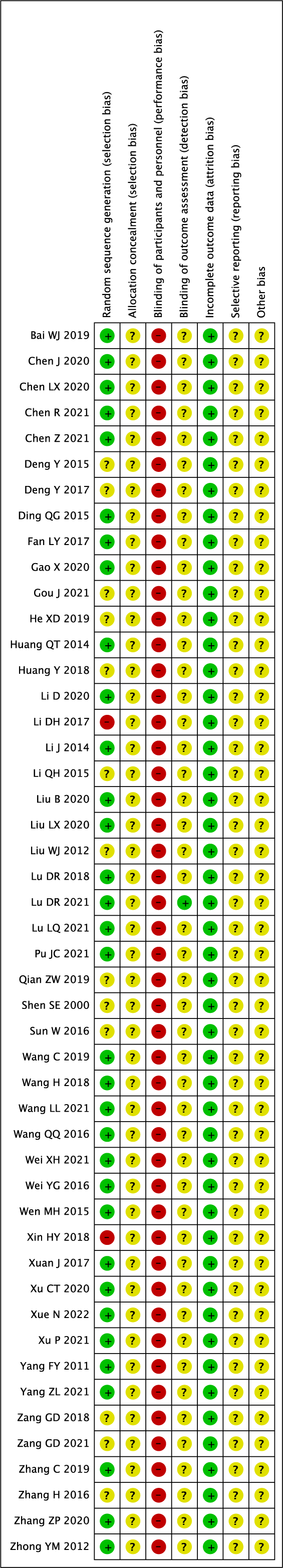

Supplement: Supplementary file 3 [file Image_2.jpeg]
